# Supplementary material for: SUMMIT: An integrative approach for better transcriptomic data imputation improves causal gene identification
Source: Nat Commun. 2022 Oct 25;13:6336. doi: 10.1038/s41467-022-34016-y (PMC9593997; doi:10.1038/s41467-022-34016-y)
Supplement: Supplementary file 1 — Supplementary Information [file 41467_2022_34016_MOESM1_ESM.pdf]

Supplementary information

SUMMIT: An integrative approach for better transcriptomic data imputation improves causal gene identification

Zichen Zhang[1], Ye Eun Bae[1], Jonathan R. Bradley[1], Lang Wu[2], Chong Wu[3,*]

[1] Department of Statistics, Florida State University, Tallahassee, FL, USA

[2] Cancer Epidemiology Division, Population Sciences in the Pacific Program, University of Hawaii Cancer Center, University of Hawaii at Manoa, Honolulu, HI, USA

[3] Department of Biostatistics, The University of Texas MD Anderson Cancer Center, Houston, TX, USA

[*] Corresponding author, Email: cwu18@mdanderson.org

September 15, 2022

Supplementary Notes

Supplementary Note 1

Here, we discuss the details on the optimization of SUMMIT.

We start with the objective function $\tilde{f}(\mathbf{w})$,

$$\tilde{f}(\mathbf{w}) = \mathbf{w}'\tilde{\mathbf{R}}\mathbf{w} - 2\mathbf{w}'\tilde{\mathbf{r}} + \theta\mathbf{w}'\mathbf{w} + J_\lambda(\mathbf{w}), \quad (1)$$

where $\mathbf{w}$ is the effect sizes of p SNPs, $\tilde{\mathbf{R}}$ is the estimated covariance matrix, $\tilde{\mathbf{r}}$ is the estimated correlation between *cis*-SNPs and the gene expression level, θ is the tuning parameter of L_2 regularization, $J_\lambda(\mathbf{w})$ is a to-be-determined penalty term with the tuning parameter λ.

The objective function above can be solved by the coordinate descent algorithm, which sequentially and iteratively solves the univariate penalized regression problem. Assume that $(\hat{w}_1^{(t)}, \ldots, \hat{w}_p^{(t)})$ is a vector of the coefficients at iteration t and we define

$$z_j^{(t)} = \tilde{r}_j - \sum_{l \neq j} \tilde{R}_{jl} \hat{w}_l^{(t)}. \tag{2}$$

By solving the univariate penalized regression problem (with $J_\lambda(w)$) given the current estimates $(\hat{w}_1^{(t)}, \ldots, \hat{w}_p^{(t)})$, one can update w_j accordingly. In the following, we will briefly describe the types of penalty we used and their corresponding updating formula.

LASSO and Elastic Net

When using the LASSO (least absolute shrinkage and selection operator)[1] and Elastic Net[2], the penalty term for w_j can be written as

$$J_\lambda(w_j) = 2\lambda((1-\alpha)|w_j| + \alpha w_j^2), \tag{3}$$

where α is a hyperparameter that controls the ratio of the L_1 and L_2 penalties. LASSO can be viewed as a special case of Elastic Net with $\alpha = 0$. For Elastic Net, we set $\alpha = 0.5$.

The updating formula for w_j is

$$\hat{w}_j^{(t+1)} = \frac{\mathcal{S}(z_j^{(t)}, \lambda(1-\alpha))}{1 + \theta + 2\alpha\lambda}, \tag{4}$$

where $\mathcal{S}(U, \lambda)$ is the soft-thresholding operator, which is defined as

$$\mathcal{S}(U,\lambda) = \begin{cases} U - \lambda, & U > \lambda; \\ U + \lambda, & U < -\lambda; \\ 0, & \text{otherwise.} \end{cases} \tag{5}$$

MCP and MNet

When using the MCP (Minimax concave penalty)[3], the penalty term for w_j can be written as

$$J_\lambda(w_j) = \begin{cases} 2(\lambda|w_j| - \frac{w_j^2}{2a}), & |w_j| \leq a\lambda; \\ a\lambda^2, & \text{otherwise.} \end{cases} \tag{6}$$

The updating formula for w_j is

$$\hat{w}_j^{(t+1)} = \begin{cases} \frac{\mathcal{S}(z_j^{(t)},\lambda)}{1+\theta-\frac{1}{a}}, & |w_j| \leq a\lambda; \\ \frac{z_j^{(t)}}{1+\theta}, & \text{otherwise.} \end{cases} \tag{7}$$

When using MNet[4], the penalty term for w_j can be written as

$$J_\lambda(w_j) = \begin{cases} 2\alpha(\lambda|w_j| - \frac{w_j^2}{2a}) + 2(1-\alpha)w_j^2, & |w_j| \leq a(\alpha\lambda)(1+(1-\alpha)\lambda); \\ a\alpha\lambda^2 + 2(1-\alpha)w_j^2, & \text{otherwise.} \end{cases} \tag{8}$$

The updating formula for w_j is

$$\hat{w}_j^{(t+1)} = \begin{cases} \frac{\mathcal{S}(z_j^{(t)},\alpha\lambda)}{1+\theta+(1-\alpha)-\frac{1}{a}}, & |w_j| \leq a(\alpha\lambda)(1+(1-\alpha)\lambda); \\ \frac{z_j^{(t)}}{1+\theta+(1-\alpha)\lambda}, & \text{otherwise.} \end{cases} \tag{9}$$

For both the MCP and MNet, we set $a = 3$. For MNet, we set $\alpha = 0.5$.

SCAD

When using the SCAD (Smoothly clipped absolute deviation)[5], the penalty term for w_j can be written as

$$J_\lambda(w_j) = \begin{cases} 2(\lambda|w_j|), & |w_j| \leq \lambda; \\ \frac{2a\lambda|w_j|-w_j^2-\lambda^2}{a-1}, & \lambda < |w_j| \leq a\lambda; \\ \lambda^2(a+1), & \text{otherwise.} \end{cases} \tag{10}$$

The corresponding updating formula for w_j is

$$\hat{w}_j^{(t+1)} = \begin{cases} \frac{\mathcal{S}(z_j^{(t)},\lambda)}{1+\theta}, & |w_j| \leq \lambda; \\ \frac{\mathcal{S}(z_j^{(t)},\frac{a}{a-1}\lambda)}{1+\theta-\frac{1}{a-1}}, & \lambda < |w_j| \leq a\lambda; \\ \frac{z_j^{(t)}}{1+\theta}, & \text{otherwise.} \end{cases} \tag{11}$$

For SCAD, we set $a = 3.7$.

Search space of the tuning parameters

We apply a warm start to generate a solution path for λ and search θ within $(0.1, 0.2, \ldots, 0.9)$. The "optimal" tuning parameters are selected based on maximizing R^2 in a tuning dataset.

Supplementary Tables

Gene	R^2	z-score	p	Trait	Method
OAS3	0.02	-4.89	1.0×10^{-6}	A2	MR-JTI
TNFSF15	0.09	4.83	1.4×10^{-6}	A2	MR-JTI
OAS3	0.02	-4.92	8.8×10^{-7}	B2	MR-JTI
CRHR1-IT1	0.43	4.71	2.5×10^{-6}	B2	TWAS-fusion
KANSL1-AS1	0.54	4.64	3.4×10^{-6}	B2	TWAS-fusion
LRRC37A4P	0.64	-4.67	3.1×10^{-6}	B2	TWAS-fusion
RP11-259G18.3	0.60	4.55	5.4×10^{-6}	B2	TWAS-fusion
RP11-707O23.5	0.54	4.73	2.2×10^{-6}	B2	TWAS-fusion
DND1P1	0.47	4.78	1.7×10^{-6}	B2	TWAS-fusion
OAS3	0.01	-4.63	3.7×10^{-6}	A2	PrediXcan
TNFSF15	0.08	4.62	3.8×10^{-6}	A2	PrediXcan
OAS3	0.01	-4.63	3.6×10^{-6}	B2	PrediXcan
OAS3	0.02	-6.00	2.0×10^{-9}	A2	UTMOST
LRRC37A	0.27	4.79	1.7×10^{-6}	B2	UTMOST
OAS3	0.02	-4.64	3.6×10^{-6}	B2	UTMOST

Supplementary Table 1: **Predicted gene expression in blood–COVID-19 associations for the likely causal genes based on the COVID-19 Host Genetics Initiative data using competing methods.** The competing methods include PrediXcan[6], TWAS-fusion[7], UTMOST[8], and MR-JTI[9]. The p-values were calculated using the two-sided Z-test. A2 represents very severe confirmed respiratory COVID-19 versus population controls. B2 represents patients hospitalized with COVID-19 versus controls. Note that for A2, TWAS-fusion identified no significant associations.

Supplementary Figures

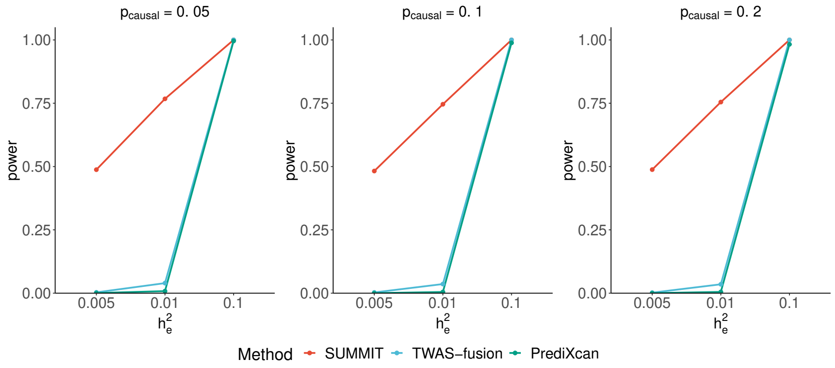

(a) $h_p^2 = 0.1$

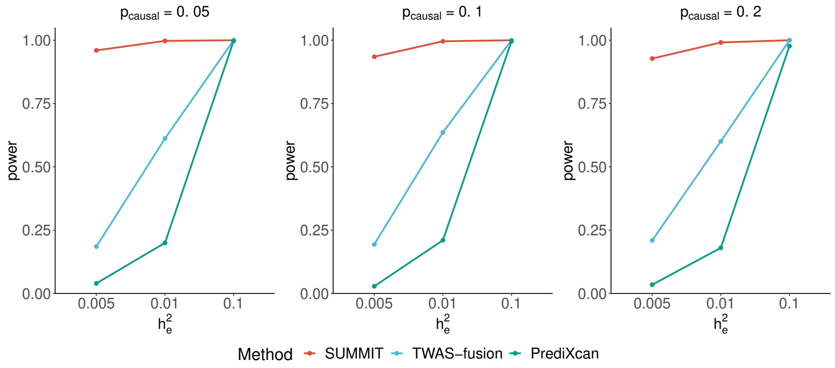

(b) $h_p^2 = 0.5$

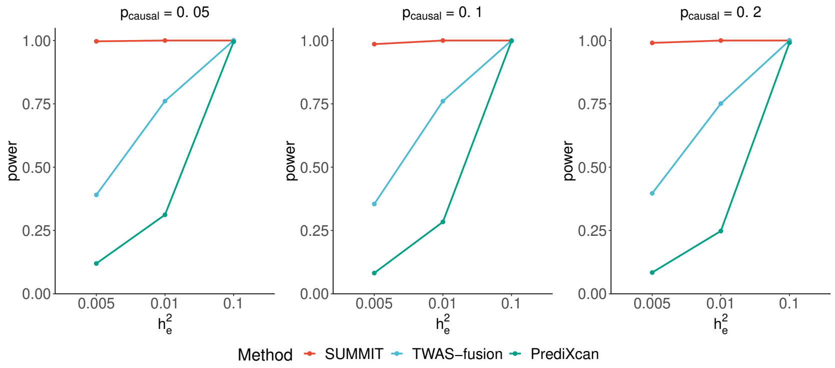

(c) $h_p^2 = 0.8$

Supplementary Figure 1: **Empirical power comparison with different h_p^2 based on the *CHURC1* gene**. SUMMIT's p-values were calculated by the SUMMIT (two-sided). TWAS-fusion's and PrediXcan's p-values were calculated using the two-sided Z-test. The empirical power was estimated by the proportions of p-values less than the significance threshold 2.5×10^{-6}.

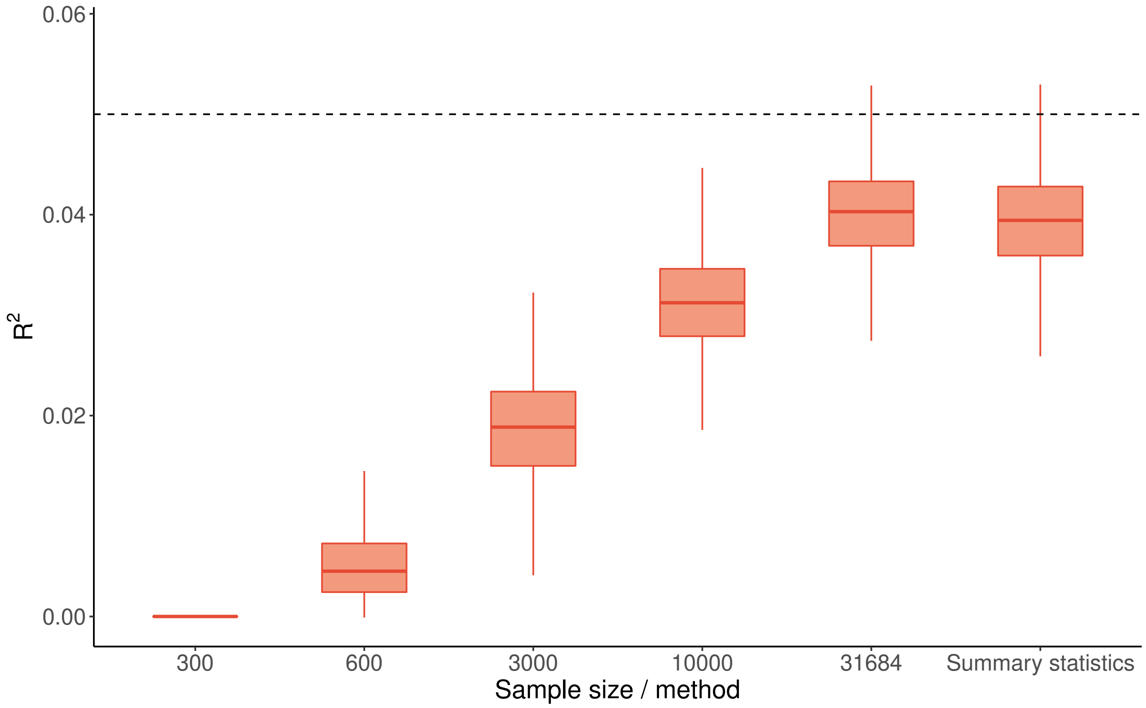

Supplementary Figure 2: **A larger training sample size increases R^2 and SUMMIT achieves the results similar to those of using individual-level data for simulations based on the *CHURC1* gene.** We set $h_e^2 = 0.05$ and $p_{causal} = 0.2$. R^2 was calculated in the testing data. Note that the "Summary statistics" boxplot was based on using simulated summary-level data with $N = 31,684$ and the other 5 boxplots' sample sizes are their corresponding ticks on the x-axis. The box limits represent the lower and upper quartiles, the central line represents the median, and the whiskers represent all samples lying within 1.5 times the interquartile range (IQR).

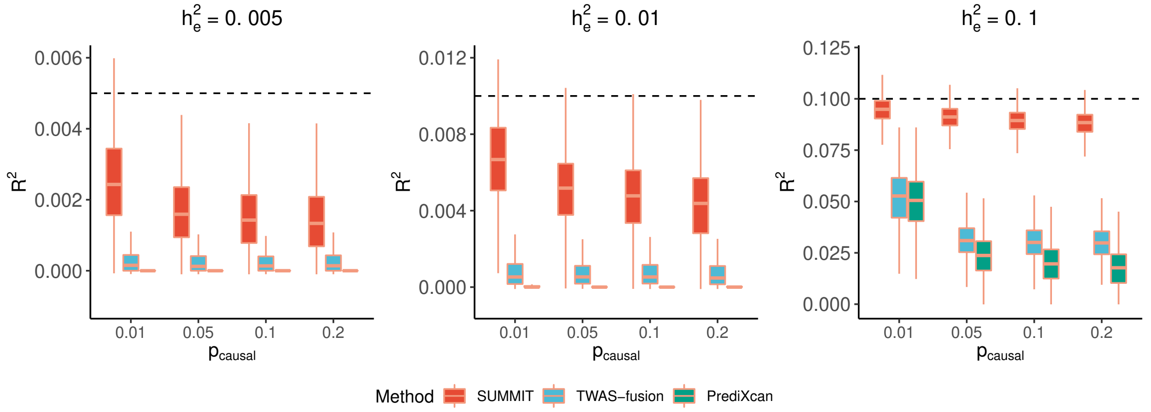

(a) Imputation R^2

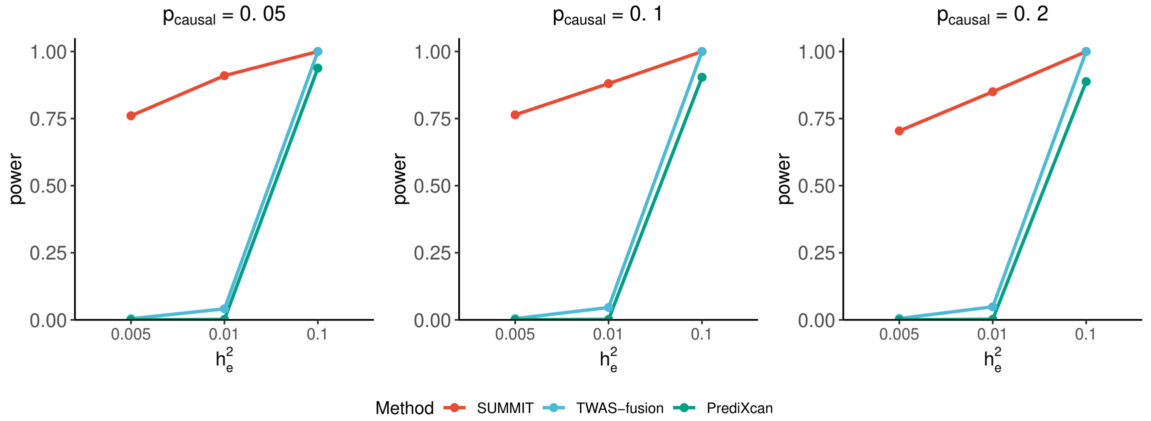

(b) Statistical power

Supplementary Figure 3: **Performance comparison in simulations based on the *ICA1* gene.** Plots of imputation R^2 (a) and subsequent power (b) in test samples by SUMMIT, TWAS-fusion, and PrediXcan, with varying true expression heritability h_e^2 and proportion of true causal SNPs p_{causal}. For (b), SUMMIT's p-values were calculated by the SUMMIT (two-sided). TWAS-fusion's and PrediXcan's p-values were calculated using the two-sided Z-test. We set $h_p^2 = 0.2$ and the empirical power was estimated by the proportions of p-values less than the significance threshold 2.5×10^{-6}. In subfigure (a), the box limits represent the lower and upper quartiles, the central line represents the median, and the whiskers represent all samples lying within 1.5 times the interquartile range (IQR); for SUMMIT, the sample size of the training data is $N = 31,684$; for both TWAS-fusion and PrediXcan, $N = 670$.

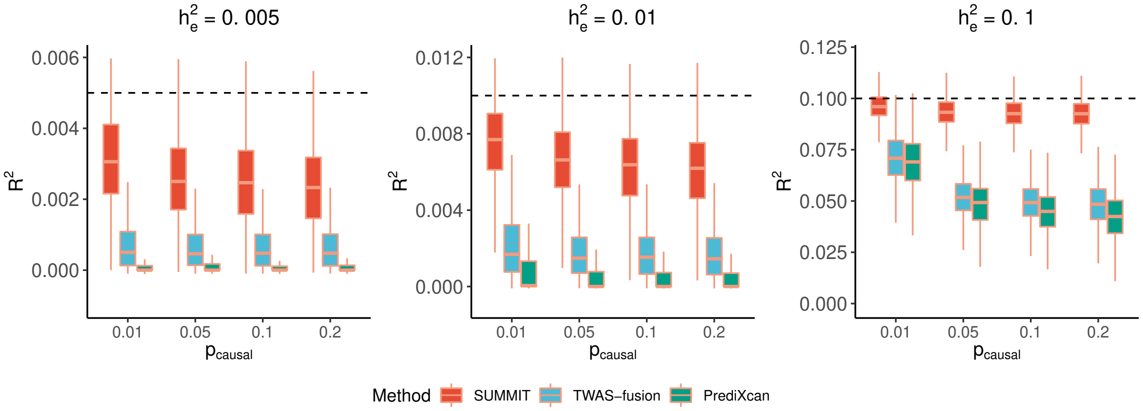

(a) Imputation R^2

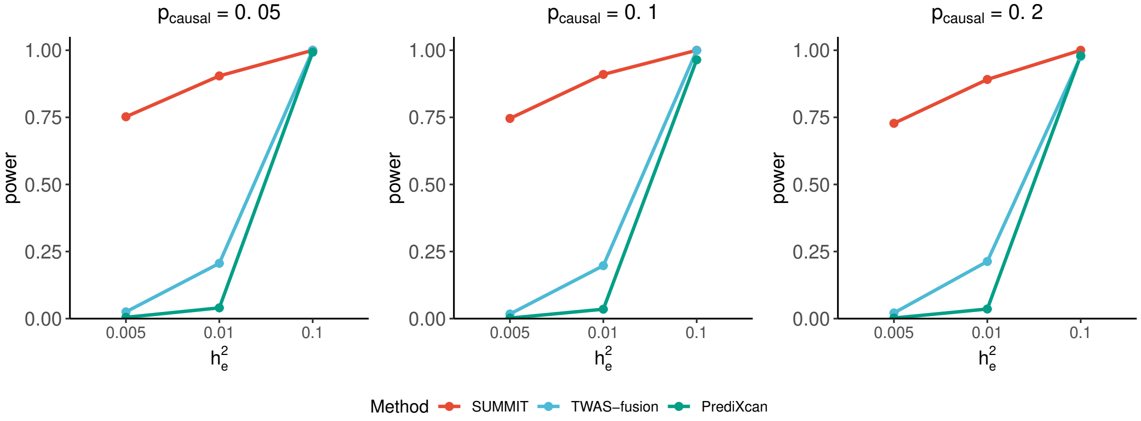

(b) Statistical power

Supplementary Figure 4: **Performance comparison in simulations based on the *KRIT1* gene.** Plots of imputation R^2 (a) and subsequent power (b) in test samples by SUMMIT, TWAS-fusion, and PrediXcan, with varying true expression heritability h_e^2 and proportion of true causal SNPs p_{causal}. For (b), SUMMIT's p-values were calculated by the SUMMIT (two-sided). TWAS-fusion's and PrediXcan's p-values were calculated using two-sided Z-test. We set $h_p^2 = 0.2$ and the empirical power was estimated by the proportions of p-values less than the significance threshold 2.5×10^{-6}. In subfigure (a), the box limits represent the lower and upper quartiles, the central line represents the median, and the whiskers represent all samples lying within 1.5 times the interquartile range (IQR); for SUMMIT, the sample size of the training data is $N = 31,684$; for both TWAS-fusion and PrediXcan, $N = 670$.

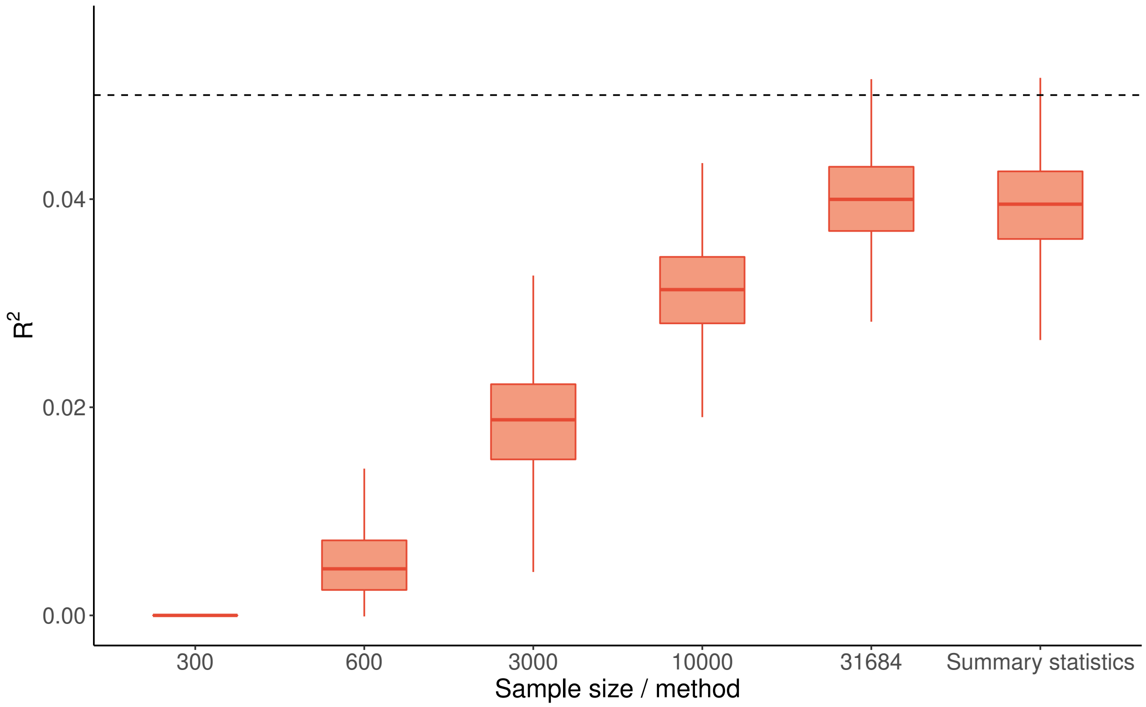

Supplementary Figure 5: **Distribution of imputation R^2 for the *ICA1* gene with respect to different sample sizes, with $h_e^2 = 0.05$ and $p_{causal} = 0.2$.** R^2 was calculated in the testing data. Note that the "Summary statistics" boxplot was based on using simulated summary-level data with $N = 31,684$ and the other 5 boxplots' sample sizes are their corresponding ticks on the x-axis. The box limits represent the lower and upper quartiles, the central line represents the median, and the whiskers represent all samples lying within 1.5 times the interquartile range (IQR).

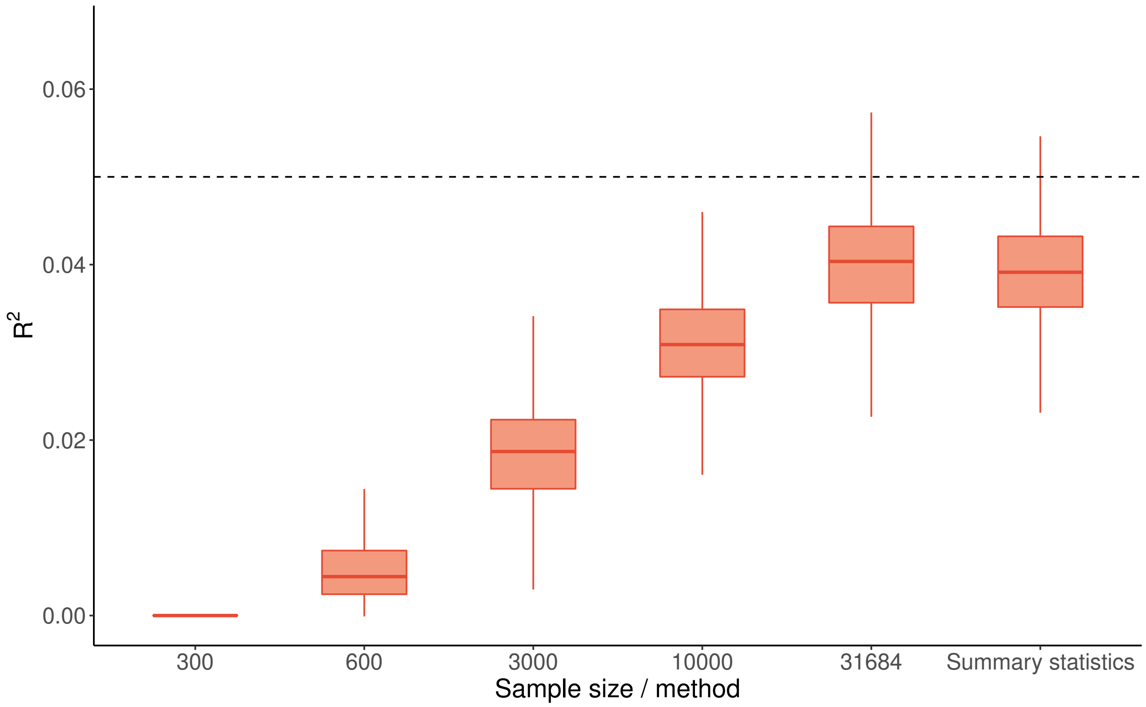

Supplementary Figure 6: **Distribution of imputation R^2 for the *KRIT1* gene with respect to different sample sizes, with $h_e^2 = 0.05$ and $p_{causal} = 0.2$.** R^2 was calculated in the testing data. Note that the "Summary statistics" boxplot was based on using simulated summary-level data with $N = 31,684$ and the other 5 boxplots' sample sizes are their corresponding ticks on the x-axis. The box limits represent the lower and upper quartiles, the central line represents the median, and the whiskers represent all samples lying within 1.5 times the interquartile range (IQR).

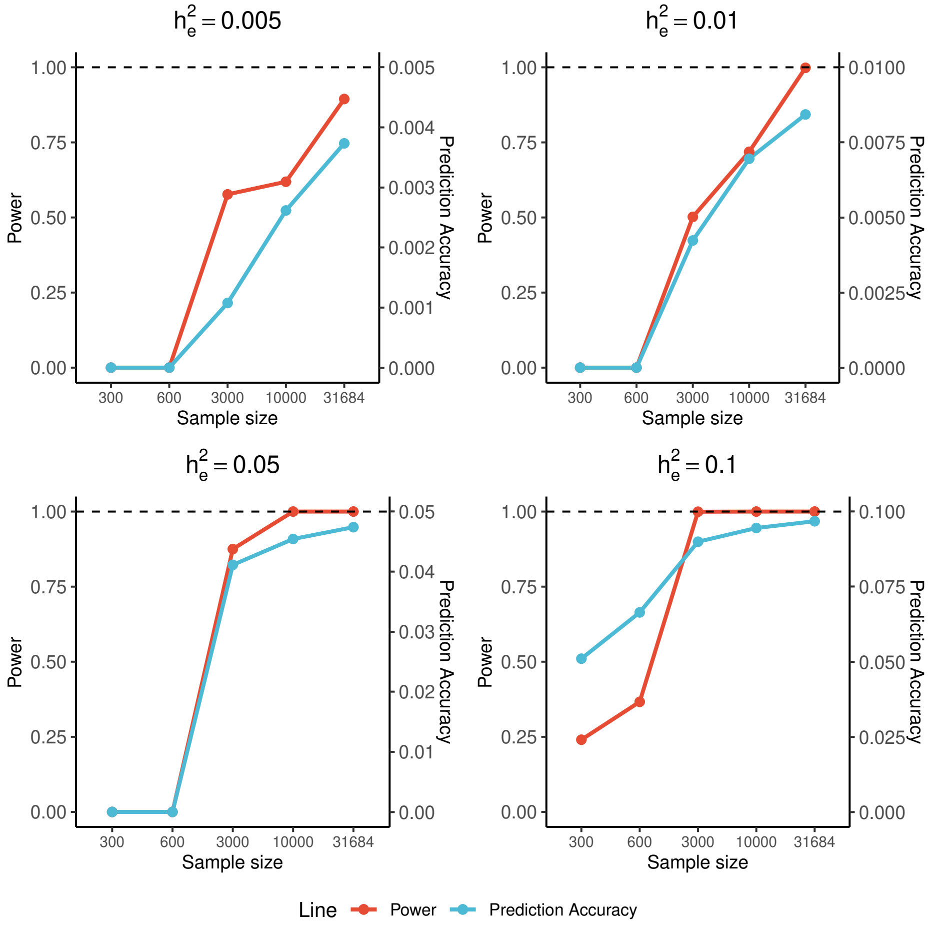

Supplementary Figure 7: **Relationship between the expression panel sample size and TWAS power or expression prediction accuracy using SUMMIT on the *CHURC1* gene with $p_{causal} = 0.01$.** The p-values were calculated by the SUMMIT (two-sided). We set $h_p^2 = 0.2$ and the empirical power was estimated by the proportions of p-values less than the significance threshold 2.5×10^{-6}.

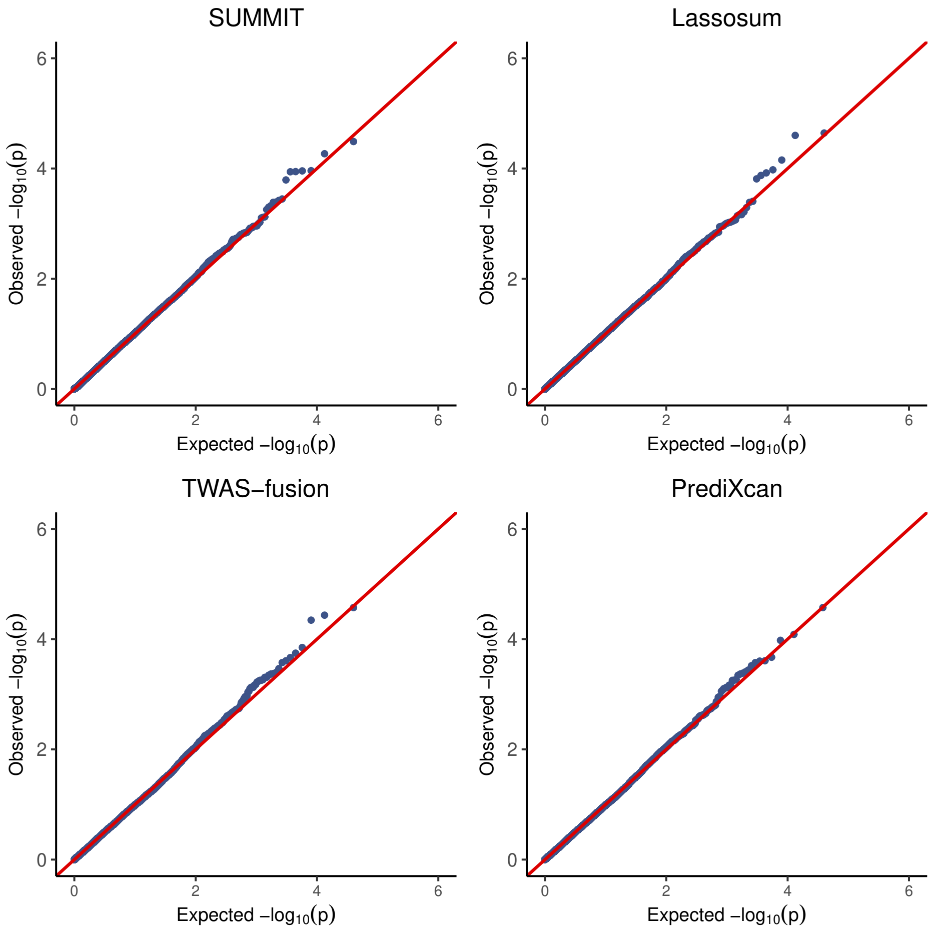

Supplementary Figure 8: **QQ plots of p-values under the null hypothesis based on the *CHURC1* gene.** We ran 5,000,000 simulations (5,000 runs for each of the 1,000 computed weights) under the null hypothesis to evaluate the Type 1 error rates. SUMMIT's p-values were calculated by the SUMMIT (two-sided). Lassosum's, TWAS-fusion's, and PrediXcan's p-values were calculated using the two-sided Z-test.

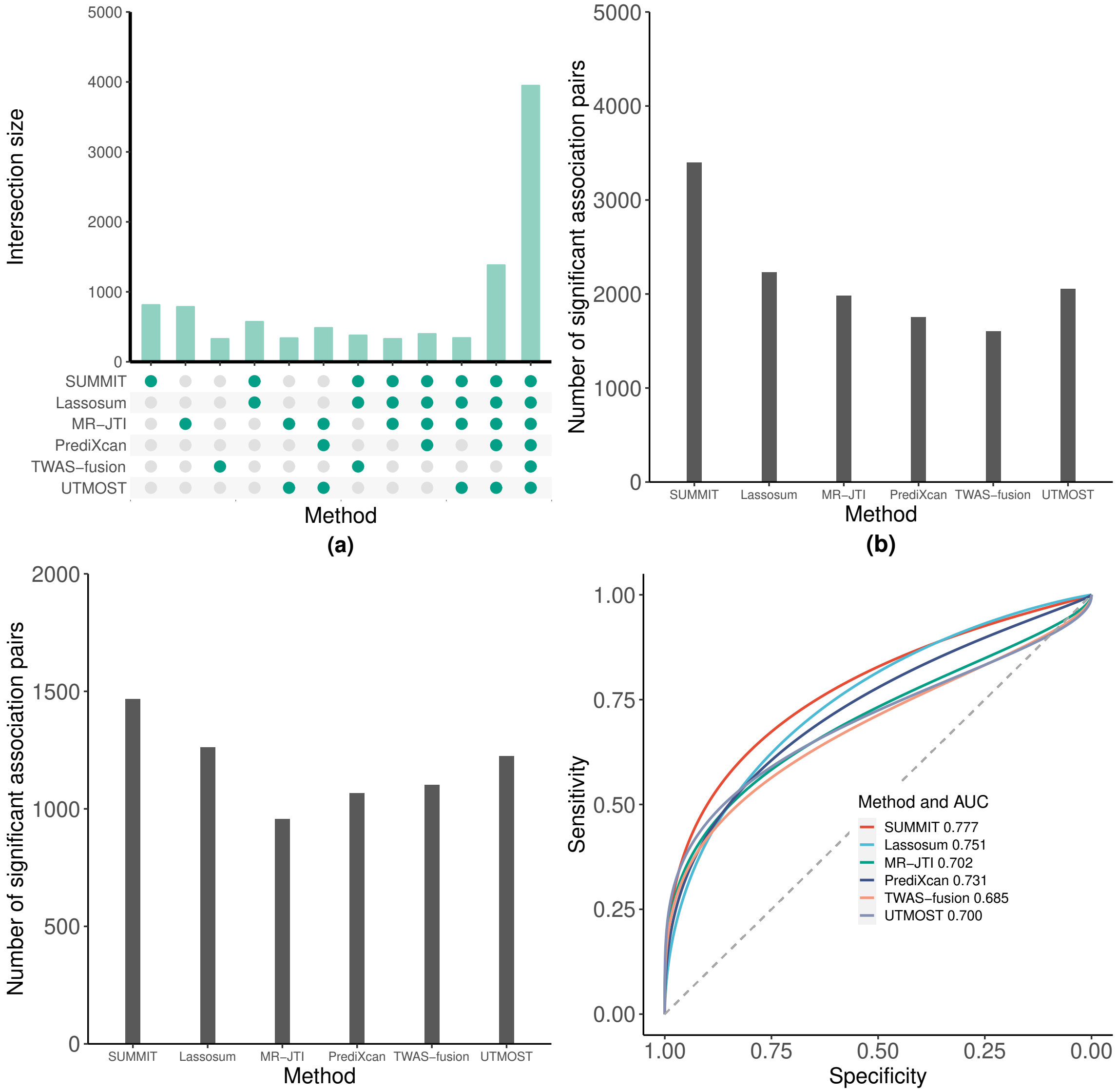

Supplementary Figure 9: **Real data results under a less stringent p-value threshold.** The settings were similar to Figure 3, except we examined SUMMIT's performance under a less stringent p-value threshold by considering the exclusion of imputation models with $0.005 \leq R^2 \leq 0.01$. (a) is the UpSet plot of overlapping imputation models with $R^2 \geq 0.01$ among different methods. (b) shows the numbers of associated genes identified by different methods when using all available genes across GWASs of 24 traits, where (c) shows the number of associated genes when evaluating a common gene set of all methods. (d) is the ROC plot for identifying "silver standard" genes.

Supplementary References

[1] Tibshirani, R. Regression shrinkage and selection via the lasso. *Journal of the Royal Statistical Society: Series B (Methodological)* **58**, 267–288 (1996).

[2] Zou, H. & Hastie, T. Regularization and variable selection via the elastic net. *Journal of the Royal Statistical Society: Series B* **67**, 301–320 (2005).

[3] Zhang, C.-H. *et al.* Nearly unbiased variable selection under minimax concave penalty. *The Annals of Statistics* **38**, 894–942 (2010).

[4] Huang, J., Breheny, P., Lee, S., Ma, S. & Zhang, C.-H. The Mnet method for variable selection. *Statistica Sinica* 903–923 (2016).

[5] Fan, J. & Li, R. Variable selection via nonconcave penalized likelihood and its oracle properties. *Journal of the American Statistical Association* **96**, 1348–1360 (2001).

[6] Gamazon, E. R. *et al.* A gene-based association method for mapping traits using reference transcriptome data. *Nature Genetics* **47**, 1091–1098 (2015).

[7] Gusev, A. *et al.* Integrative approaches for large-scale transcriptome-wide association studies. *Nature Genetics* **48**, 245–252 (2016).

[8] Hu, Y. *et al.* A statistical framework for cross-tissue transcriptome-wide association analysis. *Nature Genetics* **51**, 568–576 (2019).

[9] Zhou, D. *et al.* A unified framework for joint-tissue transcriptome-wide association and Mendelian randomization analysis. *Nature Genetics* **52**, 1239–1246 (2020).
