# Supplementary material for: SUMMIT: An integrative approach for better transcriptomic data imputation improves causal gene identification
Source: Nat Commun. 2022 Oct 25;13:6336. doi: 10.1038/s41467-022-34016-y (PMC9593997; doi:10.1038/s41467-022-34016-y)
Supplement: Supplementary file 3 — Description of Additional Supplementary Files [file 41467_2022_34016_MOESM3_ESM.pdf]

Description of Additional Supplementary Files

File Name: Supplementary Data 1

Description: The first tab contains information of 24 GWAS summary datasets (Trait, sample size, ancestry, PubMed ID, and download link). Tab 2-25 contains association studies results that we obtained using our method, SUMMIT.

File Name: Supplementary Data 2

Description: It contains a list of "silver standard" genes for each of the 24 traits used in our real data analyses.
