# Supplementary material for: SUMMIT: An integrative approach for better transcriptomic data imputation improves causal gene identification
Source: Nat Commun. 2022 Oct 25;13:6336. doi: 10.1038/s41467-022-34016-y (PMC9593997; doi:10.1038/s41467-022-34016-y)
Supplement: Supplementary file 6 — Reporting Summary [file 41467_2022_34016_MOESM6_ESM.pdf]

nature portfolio

Corresponding author(s): Chong Wu

Last updated by author(s): Aug 28, 2022

Reporting Summary

Nature Portfolio wishes to improve the reproducibility of the work that we publish. This form provides structure for consistency and transparency in reporting. For further information on Nature Portfolio policies, see our Editorial Policies and the Editorial Policy Checklist.

Statistics

For all statistical analyses, confirm that the following items are present in the figure legend, table legend, main text, or Methods section.

n/a	Confirmed	
[ ]	[x]	The exact sample size (*n*) for each experimental group/condition, given as a discrete number and unit of measurement
[x]	[ ]	A statement on whether measurements were taken from distinct samples or whether the same sample was measured repeatedly
[ ]	[x]	The statistical test(s) used AND whether they are one- or two-sided<br>*Only common tests should be described solely by name; describe more complex techniques in the Methods section.*
[x]	[ ]	A description of all covariates tested
[ ]	[x]	A description of any assumptions or corrections, such as tests of normality and adjustment for multiple comparisons
[ ]	[x]	A full description of the statistical parameters including central tendency (e.g. means) or other basic estimates (e.g. regression coefficient) AND variation (e.g. standard deviation) or associated estimates of uncertainty (e.g. confidence intervals)
[ ]	[x]	For null hypothesis testing, the test statistic (e.g. *F*, *t*, *r*) with confidence intervals, effect sizes, degrees of freedom and *P* value noted<br>*Give P values as exact values whenever suitable.*
[x]	[ ]	For Bayesian analysis, information on the choice of priors and Markov chain Monte Carlo settings
[x]	[ ]	For hierarchical and complex designs, identification of the appropriate level for tests and full reporting of outcomes
[ ]	[x]	Estimates of effect sizes (e.g. Cohen's *d*, Pearson's *r*), indicating how they were calculated

Our web collection on statistics for biologists contains articles on many of the points above.

Software and code

Policy information about availability of computer code

Data collection	No software code was used.
Data analysis	The software SUMMIT is available from https://github.com/ChongWuLab/SUMMIT.<br>The software Lassosum and the code is available from https://github.com/tshmak/lassosum.<br>The software PrediXcan and the code is available from https://github.com/hakyimlab/PrediXcan.<br>The software TWAS-fusion and the code is available from http://gusevlab.org/projects/fusion/.<br>The software Plink for handling genotype information is available from https://www.cog-genomics.org/plink/.<br>The software FOGS and the code is available from https://github.com/ChongWuLab/FOGS.<br>R (version 4.1.0) and Python 3.8 are publicly available.

For manuscripts utilizing custom algorithms or software that are central to the research but not yet described in published literature, software must be made available to editors and reviewers. We strongly encourage code deposition in a community repository (e.g. GitHub). See the Nature Portfolio guidelines for submitting code & software for further information.

Data

Policy information about availability of data

All manuscripts must include a data availability statement. This statement should provide the following information, where applicable:

- Accession codes, unique identifiers, or web links for publicly available datasets
- A description of any restrictions on data availability
- For clinical datasets or third party data, please ensure that the statement adheres to our policy

The GWAS summary data (with the download link) used in this study are summarized in Supplementary Data 2.
The eQTL summary data are available at https://www.eqtlgen.org/cis-eqtls.html.
The COVID-19 HGI summary data can be downloaded from https://www.covid19hg.org/results/.
The UK Biobank is available at https://www.ukbiobank.ac.uk/researchers/.
This research was conducted with approved access to UK Biobank data under application number 48240 https://www.ukbiobank.ac.uk/researchers/.
The genotype and RNA sequencing data for the GTEx project are available at the database of Genotypes and Phenotypes (accession number phs000424.v8.p2, https://www.gtexportal.org/home/datasets).
The processed gene expression for the GTEx project is available from the GTEx portal (https://gtexportal.org).
The MR-JTI, PrediXcan, and UTMOST models can be downloaded from https://doi.org/10.5281/zenodo.3842289.
TWAS-fusion's model can be downloaded from http://gusevlab.org/projects/fusion/.
The 1000 Genomes Project data can be downloaded from https://www.internationalgenome.org/data.
The genetic distance data for 1000 Genomes Project can be downloaded from https://github.com/joepickrell/1000-genomes-genetic-maps.
The SUMMIT models are available for free download from OSF.IO at https://doi.org/10.17605/OSF.IO/7MXSA.
The source data to replicate figures and tables in the manuscript are available from OSF.IO at https://doi.org/10.17605/OSF.IO/FJPDU.
All real data results are available at https://chongwulab.shinyapps.io/SUMMIT-app/, where practitioners can search and download results easily.

Human research participants

Policy information about studies involving human research participants and Sex and Gender in Research.

Reporting on sex and gender	does not apply
Population characteristics	does not apply
Recruitment	does not apply
Ethics oversight	does not apply

Note that full information on the approval of the study protocol must also be provided in the manuscript.

Field-specific reporting

Please select the one below that is the best fit for your research. If you are not sure, read the appropriate sections before making your selection.

☒ Life sciences ☐ Behavioural & social sciences ☐ Ecological, evolutionary & environmental sciences

For a reference copy of the document with all sections, see nature.com/documents/nr-reporting-summary-flat.pdf

Life sciences study design

All studies must disclose on these points even when the disclosure is negative.

Sample size	We used existing data that had pre-determined sample sizes. The main dataset we used is eQTLGen, which enrolled all the available blood-based eQTL datasets across 37 cohorts and obtained a total of 31,684 samples.
Data exclusions	In the eQTLGen summary datasets, we excluded SNPs that are not in the HapMap3 subset, with Minor Allele Frequency < 0.01, nonbiallelic, or ambiguous. eQTLGen is a summary-level data and we did not removed subjects from our analysis.
Replication	The final software and replication codes are made available through a Github repository (https://github.com/ChongWuLab/SUMMIT/).
Randomization	In this study, a new method, SUMMIT, was proposed, and real data analyses were conducted based on observational data (i.e., summary-level eQTL data and summary-level genome-wide association study data). This means that this study did not involve randomization.
Blinding	We used summary-level data in our data analyses, where individual-level information is summarized into several summary statistics (such as effect sizes). This means that blinding was not relevant for this study.

Reporting for specific materials, systems and methods

We require information from authors about some types of materials, experimental systems and methods used in many studies. Here, indicate whether each material, system or method listed is relevant to your study. If you are not sure if a list item applies to your research, read the appropriate section before selecting a response.

Materials & experimental systems

n/a	Involved in the study
☒	☐ Antibodies
☒	☐ Eukaryotic cell lines
☒	☐ Palaeontology and archaeology
☒	☐ Animals and other organisms
☒	☐ Clinical data
☒	☐ Dual use research of concern

Methods

n/a	Involved in the study
☒	☐ ChIP-seq
☒	☐ Flow cytometry
☒	☐ MRI-based neuroimaging
